# Mapping the generative era: a bibliometric and specialty-focused analysis of large language models in healthcare

**DOI:** 10.1186/s42492-026-00228-y

**Published:** 2026-08-14

**Authors:** Jianding Peng, Yao Tuo, Gang Wang, Ruiqin Han, Qing Wang, Yi Wang, Zixin Hu, Xiaofei Hu

**Affiliations:** 1 Beijing Tongren Hospital, Capital Medical University, Beijing, 100086 China; 2 Department of Ophthalmology, Jiangbei Campus, First Affiliated Hospital of Army Medical University, Chongqing, 400038 China; 3 Chongqing Liangjiang New Area Traditional Chinese Medicine Hospital, Chongqing, 400038 China; 4 State Key Laboratory of Common Mechanism Research for Major Diseases, Institute of Basic Medical Sciences, Chinese Academy of Medical Sciences and Peking Union Medical College, Beijing, 100086 China; 5 Institute of Medical Information, Chinese Academy of Medical Sciences & Peking Union Medical College, Beijing, 100086 China; 6 School of Computer Science, Chongqing University, Chongqing, 400000 China; 7 Artificial Intelligence Innovation and Incubation Institute, Fudan University, Shanghai, 200433 China; 8 Department of Nuclear Medicine, Southwest Hospital, Third Military Medical University (Army Medical University), Chongqing, 400038 China

**Keywords:** Large language model, Bibliometric analysis, Healthcare application, DECIDE-AI, Specialty-specific adoption (medicine, surgery, radiology)

## Abstract

Large language models (LLMs) have been rapidly adopted in healthcare since 2022; however, field-level trends and specialty differences remain poorly characterized. This study aims to map the research landscape of LLMs in healthcare and generate comparative specialty profiles through a bibliometric analysis using the Web of Science Core Collection (2015–2025), including English-language articles and reviews analyzed for publication and citation trends, leading countries, institutions, authors, journals, co-citation networks, and keyword structures with VOSviewer and CiteSpace, with sub-analyses for medicine, general and internal; surgery; and radiology, nuclear medicine and medical imaging. Of the 2226 articles included, the annual output increased from four publications in 2022 to 1327 in 2025, yielding a compound annual growth rate (CAGR) of 592.26%; the United States of America contributed 43.5% of the publications, followed by China (14.3%), Germany (9.2%), Turkey (9.0%), and England (6.8%); the publications for each specialty were surgery (31.7%), medicine, general and internal (24.2%), and radiology, nuclear medicine and medical Iimaging (13.0%), with the last demonstrating the fastest 2023–2024 growth (CAGR 265%); and the collaboration networks were United States of America-centered, with dense trans-Atlantic ties. LLM research in healthcare is expanding rapidly with distinct specialty-specific trajectories, and interpreting these trajectories using the DECIDE-AI (developmental and exploratory clinical Iinvestigations of decision support systems driven by artificial intelligence) framework clarifies low-risk near-term applications and monitoring priorities, providing a specialty-aware baseline to support the safe, equitable, and regulation-aligned adoption of LLMs in clinical practice.

**Supplementary Information:**

The online version contains supplementary material available at 10.1186/s42492-026-00228-y.

## Introduction

Healthcare systems face persistent challenges, including diagnostic errors, excessive documentation burdens, patient-communication barriers, and widening care inequities [1, 2]. Collectively, these issues limit the quality, safety, and efficiency of care delivery. Large language models (LLMs), a subset of generative artificial intelligence (GenAI), offer promising solutions by enabling scalable summarization, report generation, clinical communication, and decision support across diverse healthcare settings [3, 4]. Since late 2022, models such as GPT-4, Gemini, Claude, and Llama have demonstrated advanced capabilities in medical-question answering, documentation, and multimodal integration [5, 6]. However, their adoption has raised critical concerns regarding their fairness, toxicity, privacy, and safe deployment, particularly in regulated clinical environments [7]. For clinicians, health-system leaders, and policymakers, understanding both the volume and nature of LLM research is essential to guide implementation, set evaluation priorities, and develop governance strategies [8, 9]. Therefore, comprehensive bibliometric mapping is required to quantify research growth, identify specialty-specific trajectories, and translate bibliometric findings into actionable evaluation and governance strategies.

Growing empirical evidence highlights the transformative potential of LLMs in medical tasks. However, systematic evaluations remain disproportionately focused on question-answering performance and accuracy metrics [10]. The broader evidence base remains fragmented, with studies often limited to specific specialties or conceptual frameworks rather than providing a comprehensive field-level perspective [11, 12]. Few quantitative analyses have examined the evolution of research outputs, collaboration networks, influential journals, high-impact articles, and thematic trends over time. Domain-specific comparative analyses remain limited, thereby constraining insights into how different specialties adopt and evaluate LLMs, despite their distinct clinical tasks, data modalities, and safety profiles [13]. Importantly, few syntheses explicitly link specialty trajectories to early-stage clinical evaluation frameworks such as developmental and exploratory clinical investigations of decision support systems driven by artificial intelligence (DECIDE-AI), resulting in a gap between bibliometric signals and actionable clinical evaluations [14].

This study addresses these gaps by mapping LLM research in healthcare and focusing on specialty-specific profiles in medicine, general and internal; surgery; and radiology, nuclear medicine and medical imaging. These specialties span a range of clinical risks and modalities: medicine, general and internal emphasizes triage and patient communication; surgery focuses on perioperative education and high-stakes management; and radiology, nuclear medicine and medical imaging operates at the forefront of multimodal workflows and structured reporting. Collectively, these fields encompass diverse data types, safety thresholds, and deployment pathways critical for the early adoption of LLMs in clinical care.

Guided by pragmatic adoption signals, this study addresses three key questions. First, what are the global and temporal patterns of LLM publications in healthcare and which countries, institutions, and journals lead the field? Second, how do knowledge structures differ across specialties in terms of co-citation networks, keyword patterns, and high-impact topics? Third, which specialty-specific use cases and evaluation metrics can inform safe near-term deployment when interpreted using the DECIDE-AI framework? This study uses DECIDE-AI to interpret bibliometric patterns based on clinically relevant evaluation priorities while highlighting monitoring and reporting considerations for early-stage implementation.

## Methods

### Data sources and search strategy

A bibliometric study was conducted using the Web of Science Core Collection (WoSCC). Search queries included terms related to LLMs and the broader category of GenAI to maximize coverage, restricted to biomedical and health-related subject categories to ensure relevance. Throughout this study, ‘LLMs’ refers specifically to transformer-based language models, while “generative AI” is used when discussing the broader technological landscape. Eligible records included peer-reviewed articles and reviews published in English between January 1, 2015 and December 31, 2025. Full records with cited references were exported. The analyses encompassed annual publication and citation trends; geographic distribution by country and region; institutional contributions; authorship and collaboration; journals and dual-map overlays; highly cited publications and co-citation patterns; and keyword co-occurrence, clustering, and timelines. Sub-analyses were additionally performed for three specialties: medicine, general and internal; surgery; and radiology, nuclear medicine and medical imaging. Analytical tools included VOSviewer 1.6.19 and CiteSpace 7.0.0. Microsoft Excel was used for descriptive statistics and figure construction.

The search strategy was iteratively refined using domain-expert inputs to balance the sensitivity and specificity. The final query was as follows:

TS=(“large language model” OR “Large Language Models” OR “Generative Pre-trained Transformer” OR ChatGPT OR “GPT-3” OR “GPT-4” OR “Med-PaLM” OR BioBERT OR “Generative AI” OR “Generative Artificial Intelligence” OR GenAI).

AND TS=(medic OR health OR clinic OR patient OR biomedical OR “basic medicine” OR physiology OR pathology OR pharmacology OR surgery OR oncology OR cardiology OR neurology).

AND WC=(“Biochemistry & Molecular Biology” OR “Cell Biology” OR “Genetics & Heredity” OR “Pharmacology & Pharmacy” OR “Physiology” OR “Immunology” OR “Pathology” OR “Medicine, General & Internal” OR “Surgery” OR “Oncology” OR “Radiology, Nuclear Medicine & Medical Imaging” OR “Public, Environmental & Occupational Health”).

The search strategy included both “large language models” and “generative AI” terms to capture the full spectrum of relevant publications, though our analysis focuses primarily on LLM-specific applications in healthcare.

To provide contextual information on the rapidly emerging AI research outside the core peer-reviewed corpus, supplementary Table 1 summarizes highly cited conference publications. This table is provided for context only and was not incorporated into the main bibliometric analysis. Preprints were not included because of inconsistent indexing standards in the WoSCC and potential variability in methodological rigor, which aligns with the study’s focus on peer-validated research output.

### Inclusion and exclusion criteria

Literature screening was independently conducted by two researchers using a standardized checklist, and discrepancies were resolved through discussion with a third senior researcher to ensure consistency. Eligible records included peer-reviewed English-language articles and reviews indexed in the WoSCC under the biomedical or health categories. Non-peer-reviewed sources (editorials, letters, conference abstracts/proceedings, and preprints) and publications outside the healthcare context were excluded. Although the search period spanned 2015 to 2025, substantive outputs were concentrated between 2021 and 2025, forming the analytical focus. Duplicate records were excluded based on title/DOI matching. Keyword variants were manually normalized before co-occurrence analysis.

### Analytical framework

Analyses were conducted across seven dimensions: (1) publication and citation trajectories; (2) countries and regional-collaboration patterns; (3) institutional contributions and networks; (4) author productivity and co-citation; (5) journals and dual-map overlays; (6) highly cited publications and co-citation structures; and (7) keyword co-occurrence, clustering, and timelines. Specialty-specific sub-analyses replicated dimensions (6) and (7) to compare topic emphasis and influential sources across domains: medicine, general and internal (applications in primary care and general practice); surgery (applications in surgical specialties and procedural medicine); and radiology, nuclear medicine and medical imaging (applications in diagnostic imaging and related areas). Additionally, technology-specific keyword analysis was performed to examine the temporal emergence and specialty distribution of key technological approaches, including retrieval-augmented generation (RAG), parameter-efficient fine-tuning (PEFT)/low-rank adaptation, agentic AI, and vision-language models (VLMs). The frequencies of these terms were assessed using a supplementary analysis within the same WoSCC time window. The categories were not mutually exclusive and were intended to illustrate the temporal visibility of the selected technical paradigms.

For VOSviewer, the main settings included a minimum cluster size of 5, correlation-strength threshold of 0.1, and binary counting method for keyword co-occurrence. CiteSpace was configured with a time slice of 1 year, the top 50 cited references per slice, and pruning slice networks (Pathfinder) to optimize network visualization.

## Results

### Publication- and citation-trends analysis

**Fig. 1:**
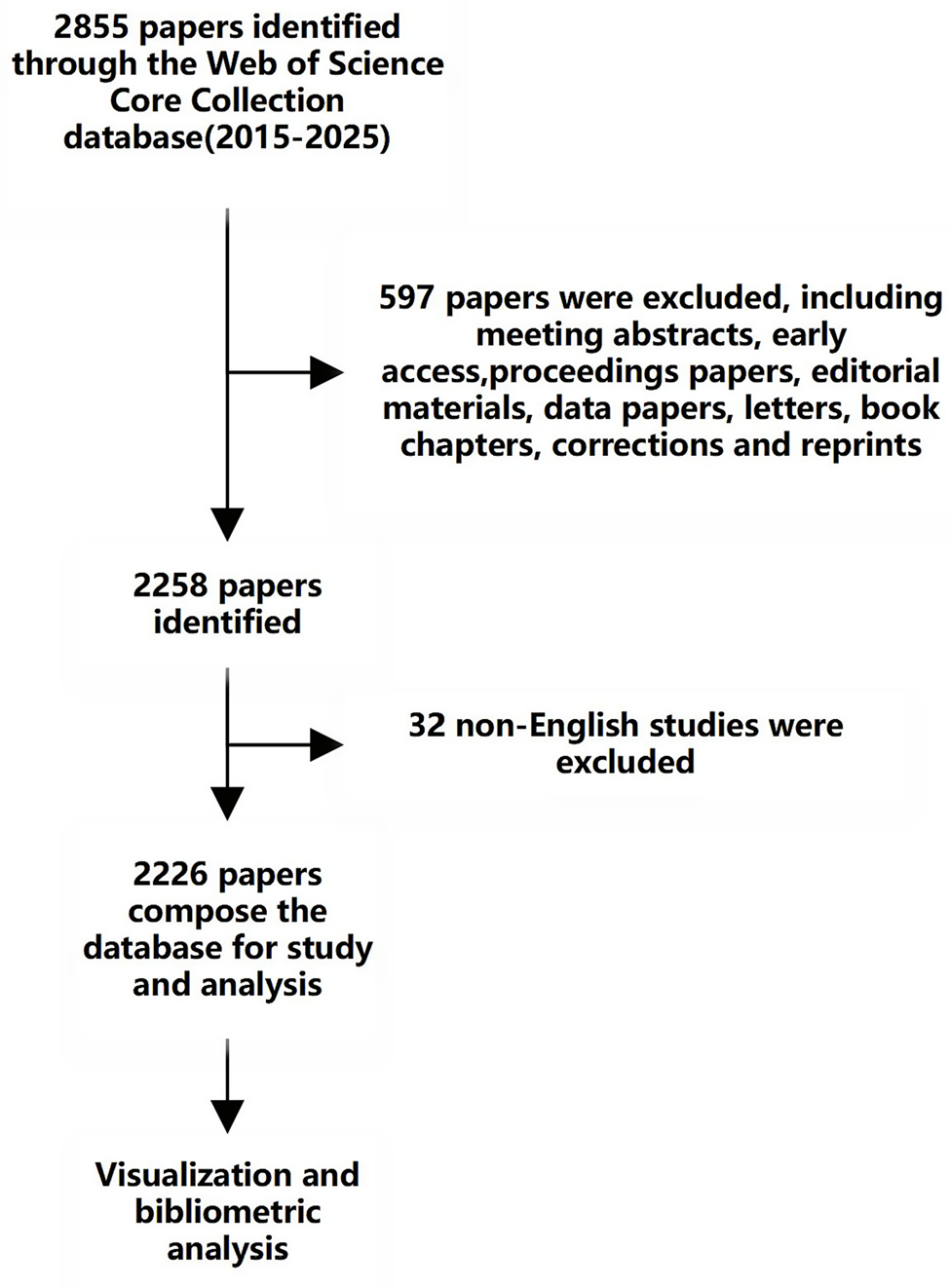
Searching strategy

**Fig. 2:**
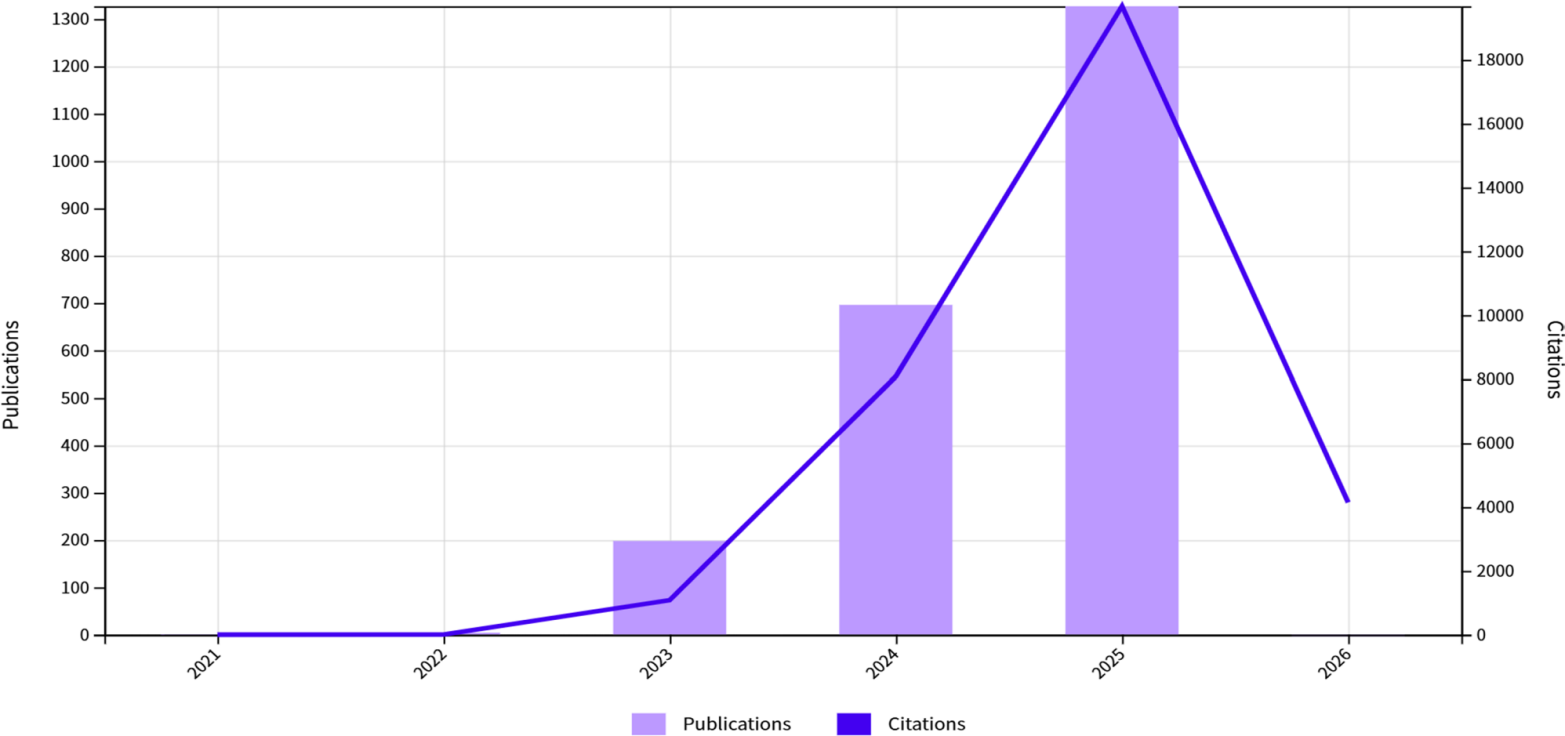
Trends in the growth of publications worldwide from 2015 to 2025. This figure illustrates the temporal evolution of publication output in large language model healthcare applications

A total of 2226 papers were included in this study (Fig. 1). Publication and citation trends from 2015 to 2025 showed minimal early activity, followed by a marked surge after 2022 (Fig. 2). No publications appeared between 2015 and 2020, and the outputs remained in below ten from 2021 to 2022. A sharp increase occurred during 2023–2025, driven by technological breakthroughs and clinical exploration, with citation patterns following similar growth.

### Geographic and regional analysis

**Fig. 3:**
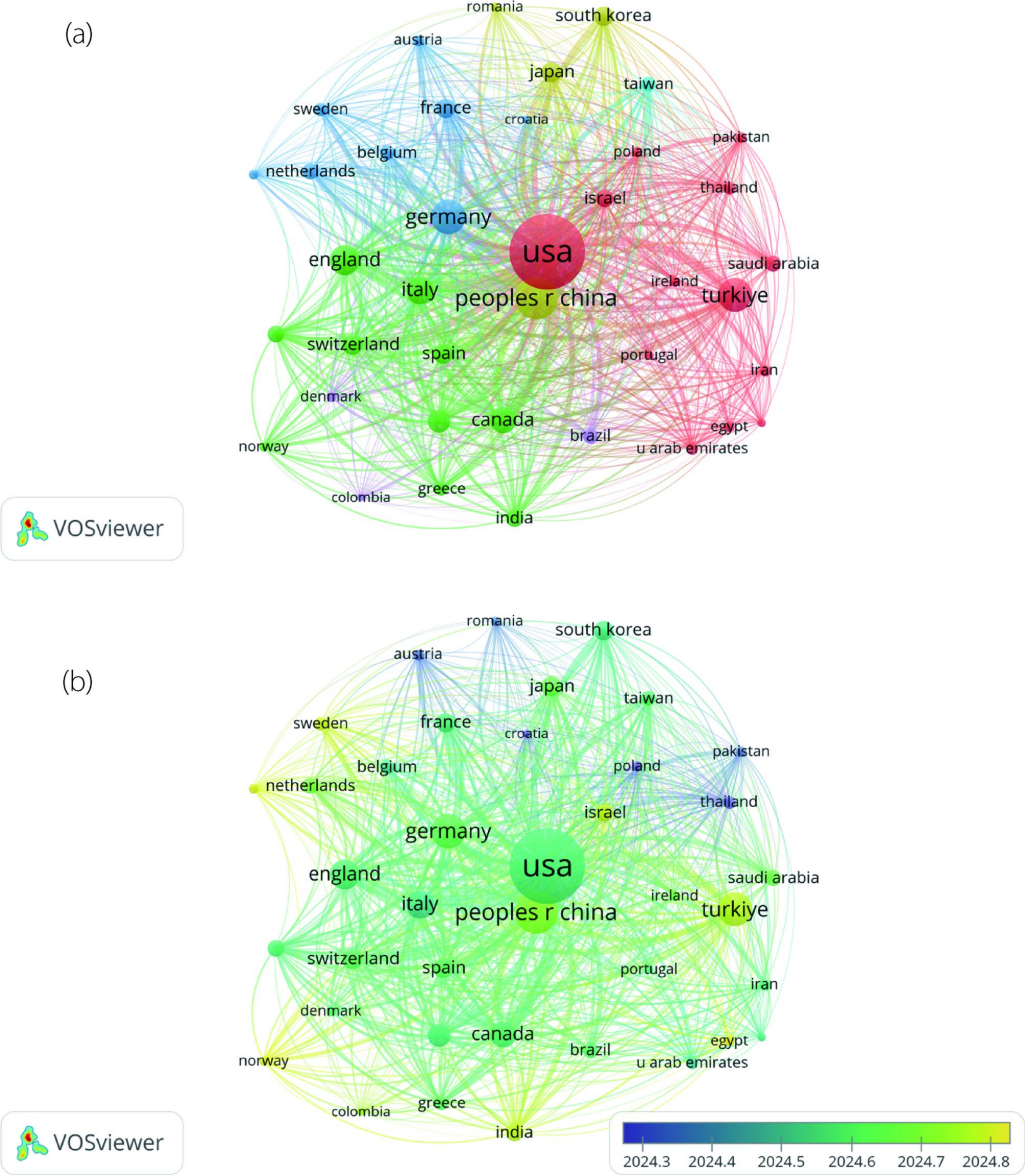
Country analyses in the field of large language models in healthcare. **a** Country-collaboration map by VOSviewer showing the global research network, with node sizes representing publication volume and connecting lines indicating collaborative relationships; **b** Time-overlapping analysis of countries and regions, with color coding representing the average publications year

The dataset included 105 countries and regions; however, only 7 nations published more than 100 articles (Fig. 3). United States of America was in the lead with 967 publications, followed by China (317), Germany (205), Turkey (201), and England (152).

The overlay visualization illustrates the average publication year for each country (Fig. 3). Owing to the limited number of publications between 2021 and 2023, the average number of publications was clustered between March and August 2024. United States of America and Germany had earlier average publication years (mid-2024), reflecting their early technological advantages and sustained research investments. By contrast, China occupied an intermediate position in terms of average publication year, with a sharp surge in output in 2024 reflecting the impact of national GenAI and health-data governance policies. Combined with the temporal-overlay analysis, this reveals a striking surge in China’s output during 2024, marking particularly rapid growth. Turkey and India exhibited later average publication years (late 2024), indicating more recent entry into the field.

### Institutional analysis

**Fig. 4:**
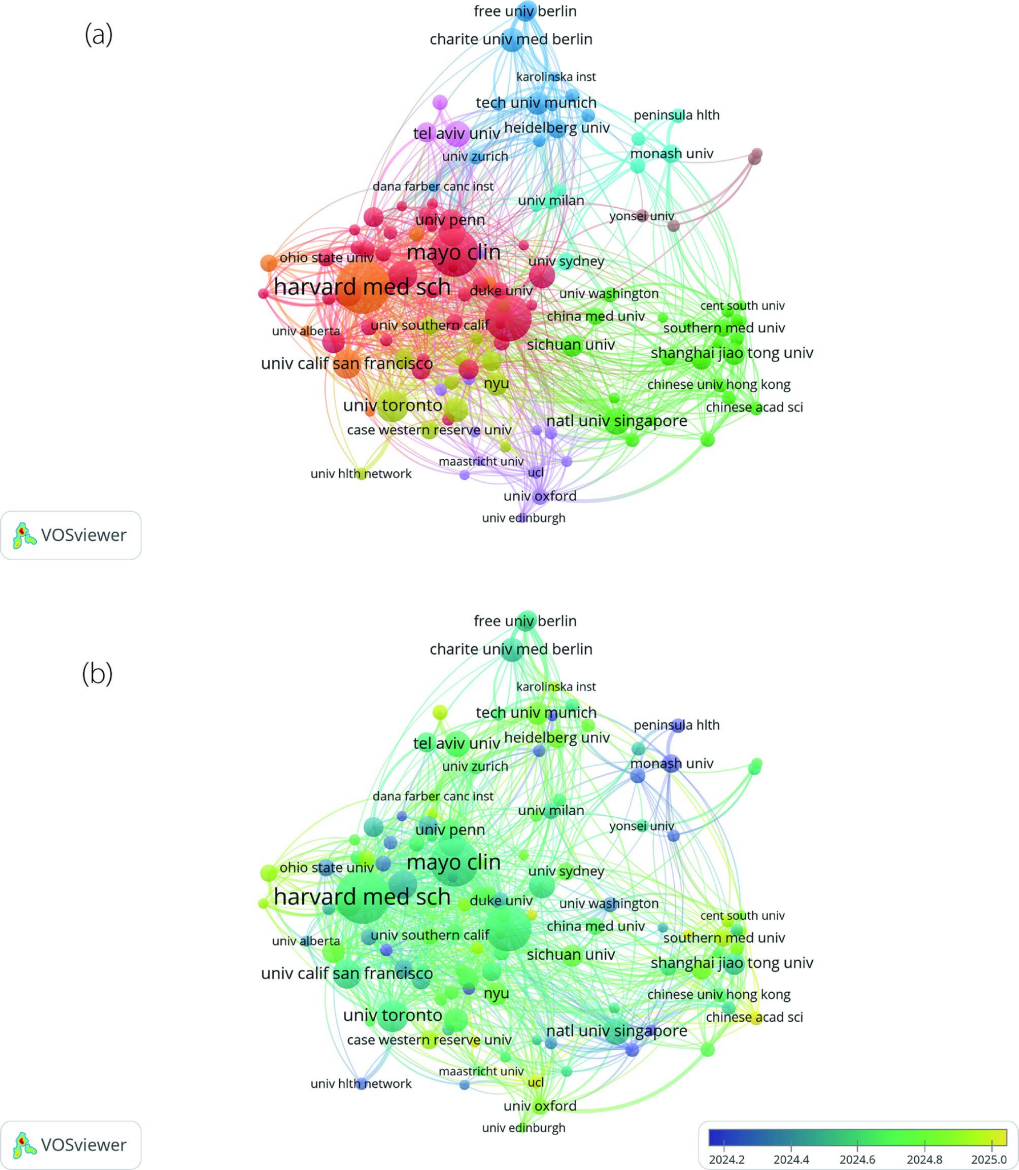
Institution analyses in the field of large language models in healthcare. **a** The collaboration network of institutions showing interconnections between research organizations; **b** Institutional-density visualization

The institutional network included 3451 institutions. Harvard Medical School (83 publications), Mayo Clinic (70 publications), and Stanford University (68 publications) were the top contributors. Stanford University led in the number of citations (4612), followed by Harvard Medical School (1541) and the National University of Singapore (801). The network exhibited a United States of America-centered hub-and-spoke structure (Fig. 4).

### Author analysis

**Fig. 5:**
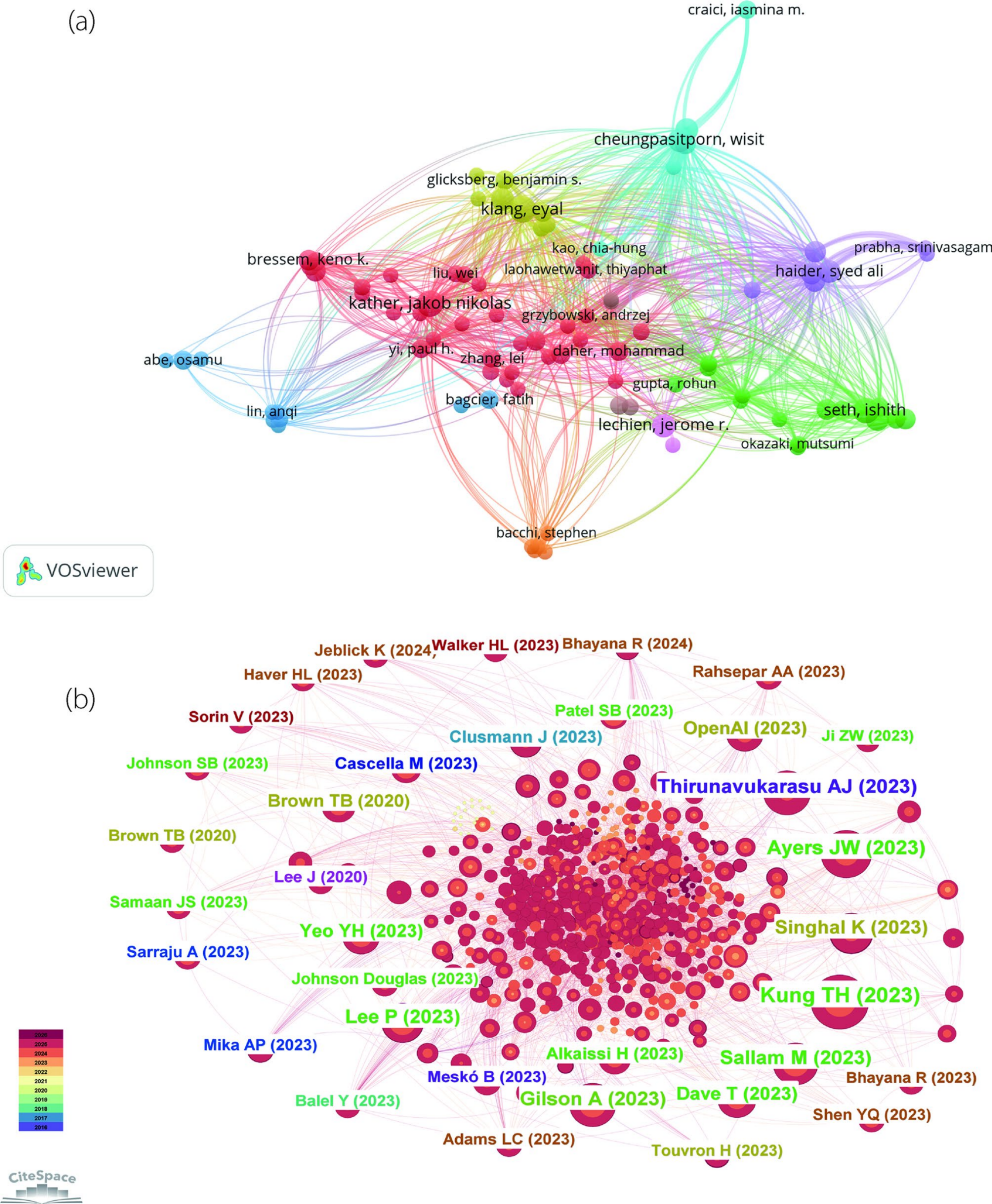
Author analyses in the field of large language models in healthcare. **a** VOSviewer overlay visualization of authors showing collaborative networks, with color coding indicating temporal patterns of publication; **b** Network of co-cited authors by CiteSpace, revealing the most influential researchers in the field

Co-citation analysis identified the most influential contributors to the field. The three most frequently co-cited authors were Kung (277), Sallam (168), and OpenAI (156). Collaboration networks revealed distinct research communities with varying levels of interconnection (Fig. 5).

### Journal analysis

**Fig. 6:**
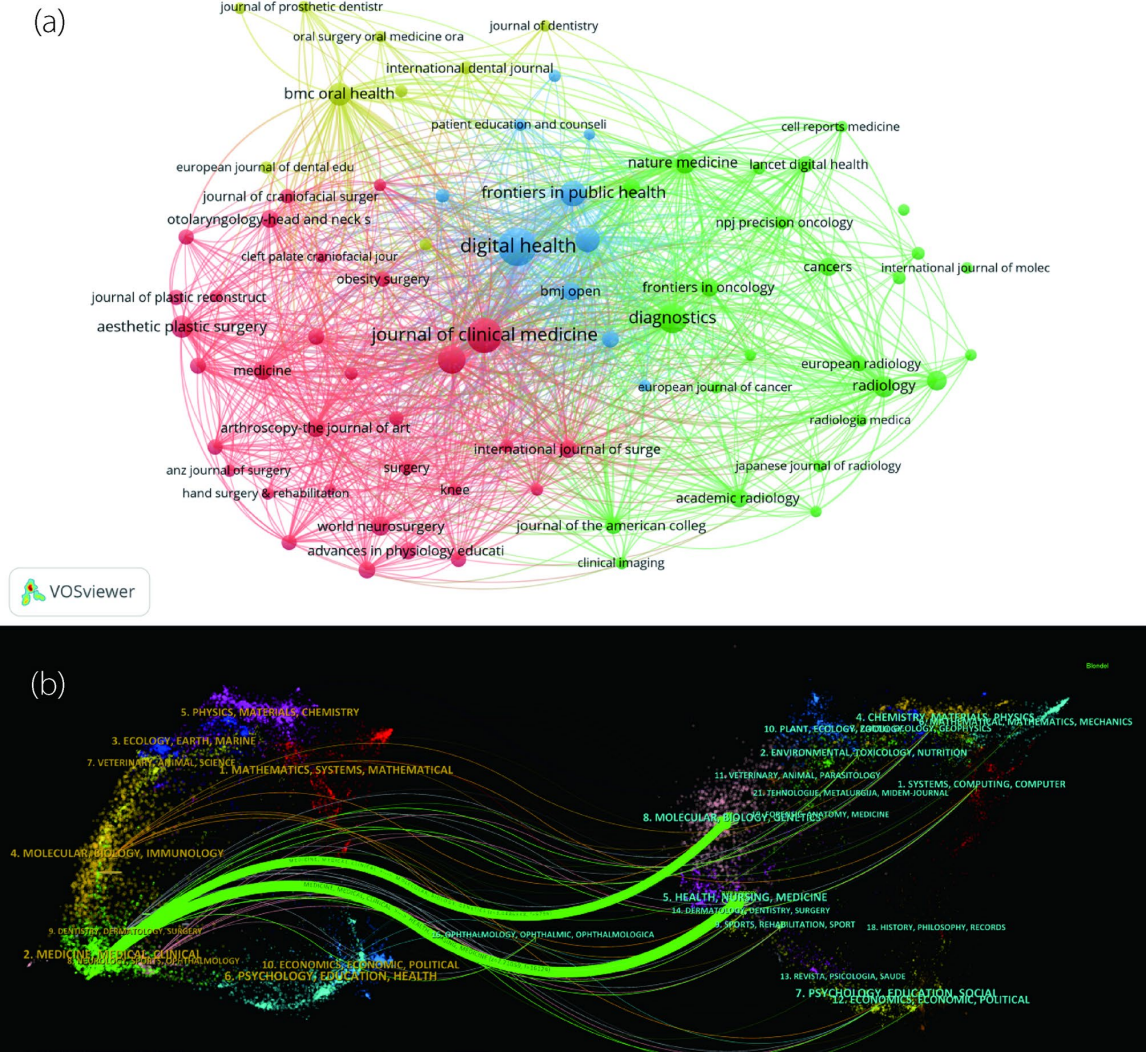
Journal and disciplinary analysis in the field of large language models in healthcare. **a** VOSviewer density visualization of journals showing publication concentration in high-impact medical journals; **b** Dual-map overlay of journals, with connecting lines demonstrating interdisciplinary knowledge transfer

Journal mapping revealed that *Nature Medicine*, *JAMA Network Open*, and *Radiology* were the leading publication platforms (Fig. 6a) alongside high-impact general medical journals such as *Frontiers in Public Health* and *Journal of Clinical Medicine*. These journals have made high-quality contributions through rigorous peer review and timely engagements with emerging research.

The dual-map overlay shows strong interdisciplinary citation flows between computer science/AI and medicine/healthcare (Fig. 6b), reflecting the reciprocal relationship between technical innovation and clinical application.

### Citation analysis

**Fig. 7:**
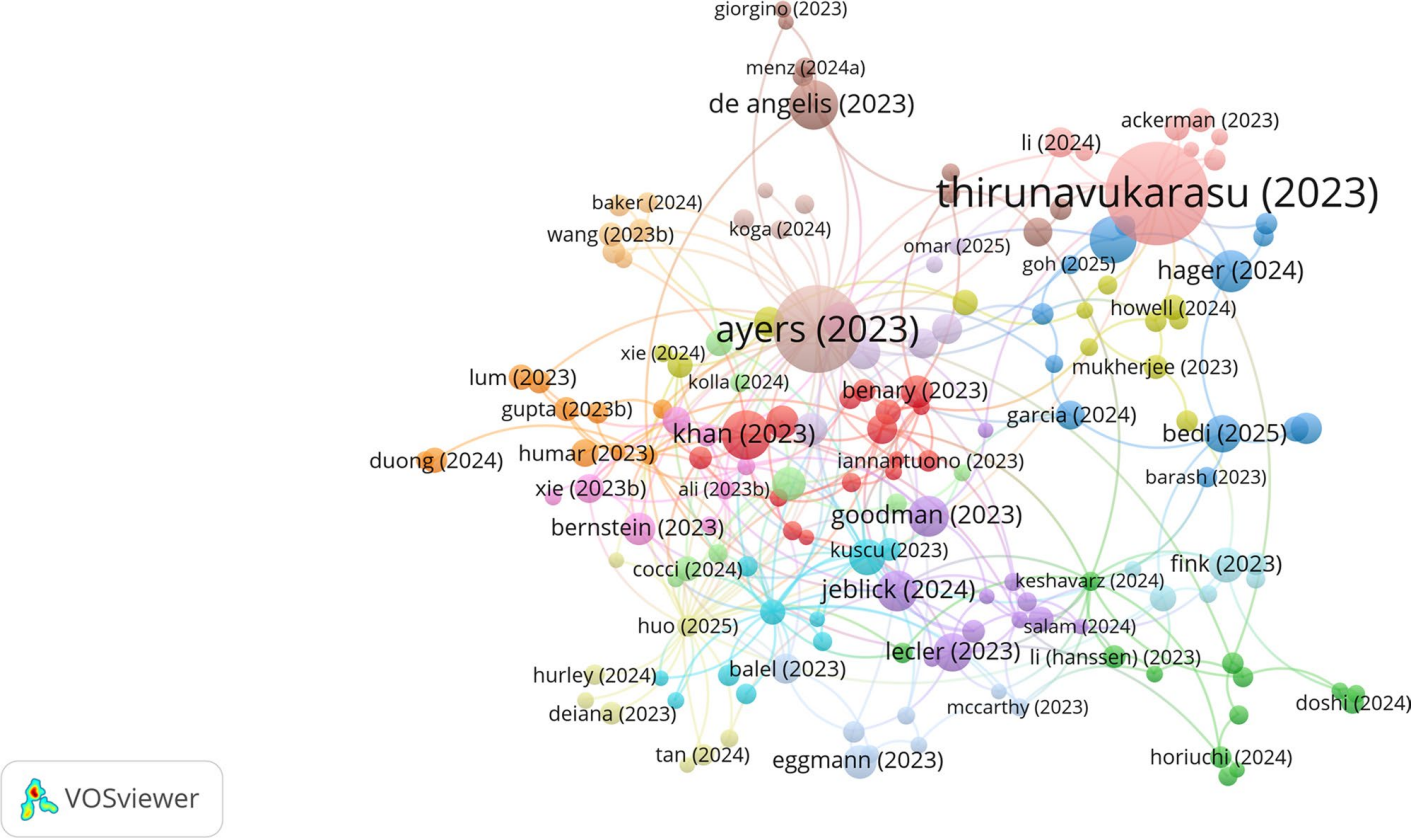
Reference analyses in the field of large language models in healthcare. Co-cited networks of references displaying the intellectual structure of the field, with clusters representing different research themes and highly cited papers serving as foundational knowledge nodes

The CiteSpace analysis identified the most highly cited publications (Fig. 7, Table 1). The top-cited article was “Large language models in medicine” by Thirunavukarasu et al., published in *Nature Medicine* (2023, 2129 citations) [3]. Other frequently cited works included “Comparing physician and artificial intelligence chatbot responses to patient questions posted to a public social media forum” by Ayers et al. (*JAMA Internal Medicine*, 2023, 1466 citations) [15], “ChatGPT and the rise of large language models: the new AI-driven infodemic threat in public health” by De Angelis et al. (*Frontiers in Public Health*, 2023, 419 citations) [16], “ChatGPT-Reshaping medical education and clinical management” by Khan and Irfan (*Pakistan Journal of Medical Sciences*, 2023, 416 citations) [17], and other foundational studies. These articles shaped the intellectual structure of the field by providing early evidence of the capabilities and limitations of LLMs in a medical context (Table 1).

**Table 1:** Top 10 highly cited publications in the field of large language models in healthcare

Rank	Title	Journal	First author	Year	Citation (total)
1	Large language models in medicine	*Nature Medicine*	Thirunavukarasu AJ	2023	2129
2	Comparing physician and artificial intelligence chatbot responses to patient questions posted to a public social media forum	*JAMA* * Internal Medicine*	Ayers JW	2023	1466
3	ChatGPT and the rise of large language models: the new AI-driven infodemic threat in public health	*Frontiers in Public Health*	De Angelis L	2023	419
4	ChatGPT-Reshaping medical education and clinical management	*Pakistan Journal of Medical Sciences*	Khan RA	2023	416
5	Adapted large language models can outperform medical experts in clinical text summarization	*Nature Medicine*	Van Veen D	2024	376
6	Evaluation and mitigation of the limitations of large language models in clinical decision-making	*Nature Medicine*	Hager P	2024	305
7	Accuracy and reliability of chatbot responses to physician questions	JAMA *Network Open*	Goodman RS	2023	290
8	ChatGPT makes medicine easy to swallow: an exploratory case study on simplified radiology reports	*European Radiology*	Jeblick K	2024	281
9	Revolutionizing radiology with GPT-based models: current applications, future possibilities and limitations of ChatGPT	*Diagnostic and Interventional Imaging*	Lecler A	2023	257
10	Testing and evaluation of health care applications of large language models: a systematic review	*JAMA-Journal of the American Medical Association*	Bedi S	2025	242

This table presents the most influential publications ranked by citation count

### **Keyword analysis**

**Fig. 8:**
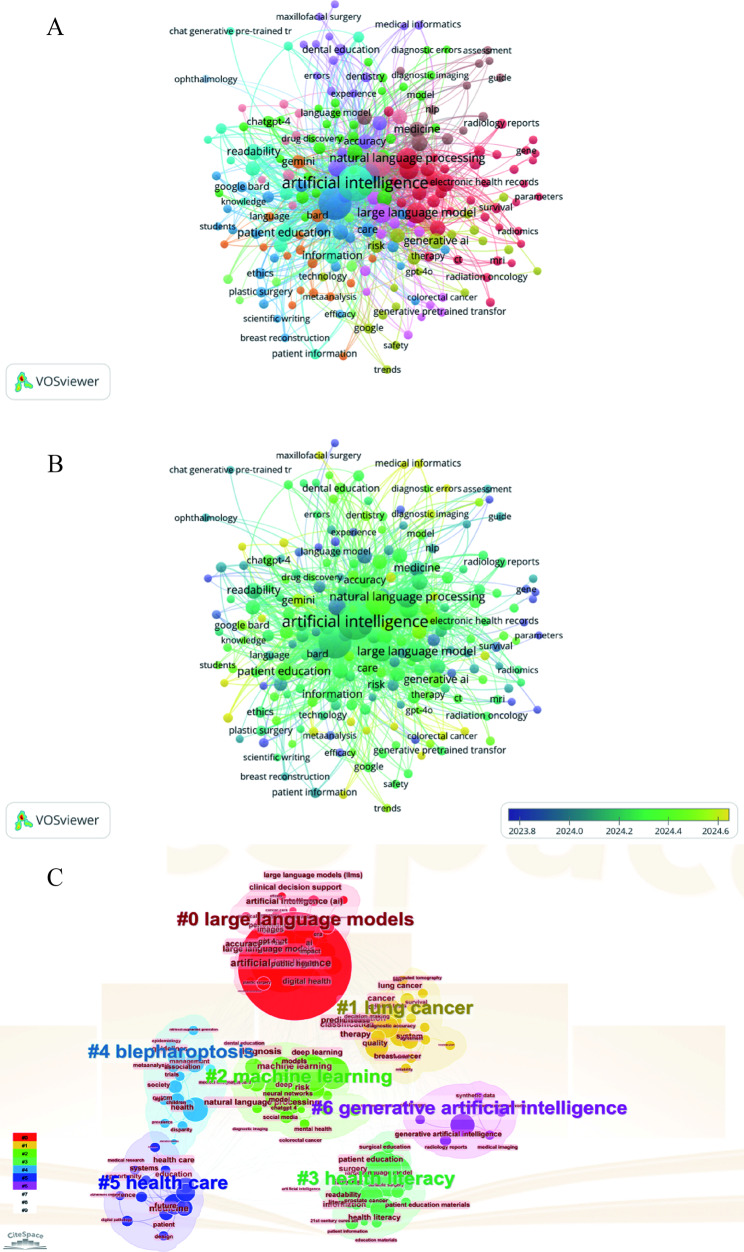
Keyword co-occurrence and trends analysis. **a** VOSviewer overlay visualization of keywords with color coding representing temporal emergence patterns; **b** Cluster diagrams of references revealing thematic groupings; **c** Timeline distribution of cluster analysis showing the chronological development of research themes

The keyword analysis captured the conceptual foundations of the current research (Fig. 8). High-frequency terms included “artificial intelligence” (757 occurrences), “large language models” (251 occurrences), and “ChatGPT” (89 occurrences). Temporal distribution indicated a recent rise in terms such as “generative artificial intelligence”.

Cluster analysis further illustrated the evolution of the research themes. The major clusters included “large language models” (#0), “lung cancer” (#1), and “machine learning” (#2). Additional clusters comprised “health literacy” (#3), “blepharoptosis” (#4), “health care” (#5), and “generative artificial intelligence” (#6), reflecting the breadth of LLM applications in healthcare.

### Technology-specific keyword analysis

Supplementary keyword analysis (Table 2) suggested a shift from general-purpose model evaluation (2022–2023) to domain-adapted and workflow-oriented applications (2024–2025). RAG-related terms (60 publications) appeared only between 2024 and 2025, predominantly in radiology and internal medicine. PEFT terms (59 publications) increased steadily after 2023 across all specialties. VLM-related studies (241 publications) were the most prominent in radiology, whereas agentic AI (16 publications) remained an emerging direction in medicine, general and internal. Collectively, technology adoption was closely related to specialty-specific clinical needs and data modalities, rather than technological advancement alone.

**Table 2:** Publication frequency and temporal distribution of technology-specific terms

Technology category	Search strategy	2022	2023	2024	2025	Total	Primary specialty
RAG	TS=(“RAG” OR “retrieval augmented generation” OR “retrieval-augmented generation” OR “retrieval augmented” OR “retrieval-grounded” OR “grounded generation” OR “retrieval-based generation”)	0	0	11	49	60	Radiology, medicine
Fine-tuning	TS=(“fine-tuning” OR “fine tuning” OR “finetuning” OR “LoRA” OR “low-rank adaptation” OR “PEFT” OR “parameter-efficient fine-tuning” OR “parameter efficient fine-tuning”)	0	1	14	44	59	All
Agentic AI	TS=(“agentic AI” OR “AI agent” OR “AI agents” OR “autonomous agent” OR “clinical agent” OR “medical agent” OR “LLM agent” OR “workflow agent” OR “multi-agent”)	0	0	2	14	16	Medicine
VLM	TS=(“vision-language model” OR “vision language model” OR “VLM” OR “VLMs” OR “multimodal large language model” OR “multimodal LLM” OR “MLLM” OR “GPT-4V” OR “GPT-4o” OR “medical vision-language model” OR “multimodal”)	0	6	38	197	241	Radiology
Medical LLMs	TS=(“large language model” OR “Large Language Models” OR “Generative Pre-trained Transformer” OR ChatGPT OR “GPT-3” OR “GPT-4” OR “Med-PaLM” OR BioBERT OR “Generative AI” OR “Generative Artificial Intelligence” OR GenAI) AND (medic OR health OR clinic OR patient OR biomedical OR “basic medicine” OR physiology OR pathology OR pharmacology OR surgery OR oncology OR cardiology OR neurology)	4	198	696	1327	2225	Medicine

### Disciplinary analysis

For the disciplinary analysis, a consistent search strategy was applied across the three Web of Science categories, varying only the subject category to target medicine, general and internal; surgery; and radiology, nuclear medicine and medical imaging.

### Medicine, general and internal field

**Fig. 9:**
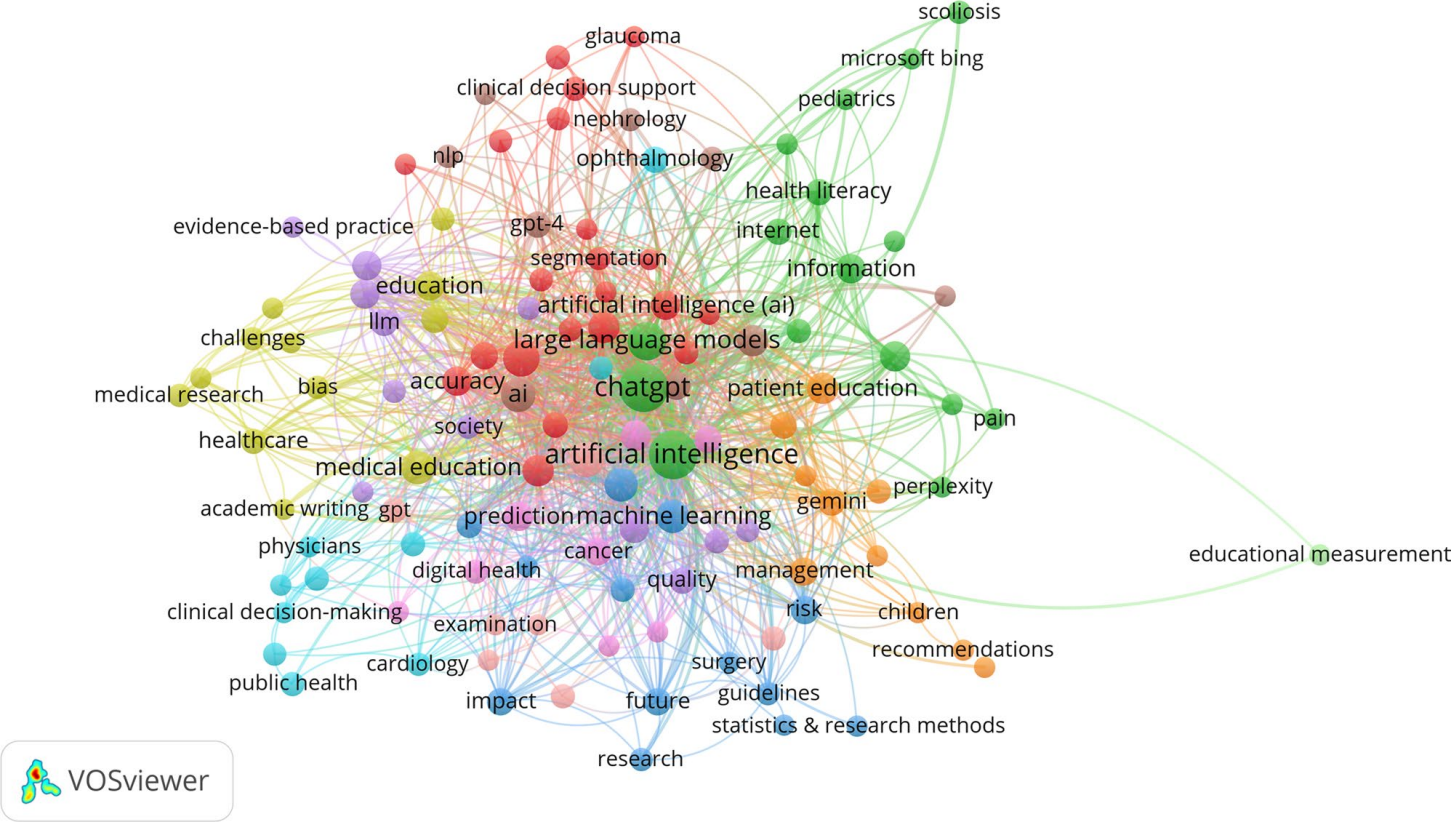
Keyword co-occurrence and trends analysis in the medicine, general and internal field

This domain yielded a total of 475 articles. Keyword co-occurrence analysis within this specialty subgroup showed a strong technology-driven orientation, with “artificial intelligence” as the most frequent term (757 occurrences), followed by “large language models” (251 occurrences) and “natural language processing” (116 occurrences) (Fig. 9, Table 3). These patterns highlight the central role of AI tools in supporting clinical decisions, data analysis, and medical education.

**Table 3:** Top 10 highly cited publications in the medicine, general and internal field

Rank	Title	Journal	First author	Year	Citation (total)
1	Comparing physician and artificial intelligence chatbot responses to patient questions posted to a public social media forum	*JAMA Internal Medicine*	Ayers JW	2023	1466
2	ChatGPT-reshaping medical education and clinical management	*Pakistan Journal of Medical Sciences *	Khan RA	2023	416
3	Accuracy and reliability of chatbot responses to physician questions	*JAMA Network Open*	Goodman RS	2023	290
4	Testing and evaluation of health care applications of large language models: a systematic review	*JAMA-Journal of the American Medical Association*	Bedi S	2025	242
5	Benchmarking large language models’ performances for myopia care: a comparative analysis of ChatGPT-3.5, ChatGPT-4.0, and Google Bard	*EBioMedicine*	Lim ZW	2023	239
6	Large language models in medicine: the potentials and pitfalls a narrative review	*Annals of Internal Medicine*	Omiye JA	2024	193
7	Leveraging large language models for decision support in personalized oncology	*JAMA Network Open*	Benary M	2023	179
8	Comparison of ophthalmologist and large language model chatbot responses to online patient eye care questions	*JAMA Network Open*	Bernstein IA	2023	170
9	FDA perspective on the regulation of artificial intelligence in health care and biomedicine	*JAMA-Journal of the American Medical Association*	Warraich HJ	2025	161
10	Generative artificial intelligence to transform inpatient discharge summaries to patient-friendly language and format	*JAMA Network Open*	Zaretsky J	2024	152

Highly cited studies emphasized both technological validation and ethical considerations. For example, Ayers et al. (2023, *JAMA Internal Medicine*, 1466 citations) [15] showed that ChatGPT performed comparably to junior physicians in routine consultations, but exhibited reasoning gaps in complex cases. Other influential studies, such as those by Khan and Irfan (2023, *Pakistan Journal of Medical Sciences*, 416 citations) [17] and Bedi et al. (2025, *JAMA*, 242 citations) [18], further explored LLM applications in medical education and healthcare-system evaluation, underscoring the need for human-AI collaboration to balance efficiency and safety.

#### Surgery field

**Fig. 10:**
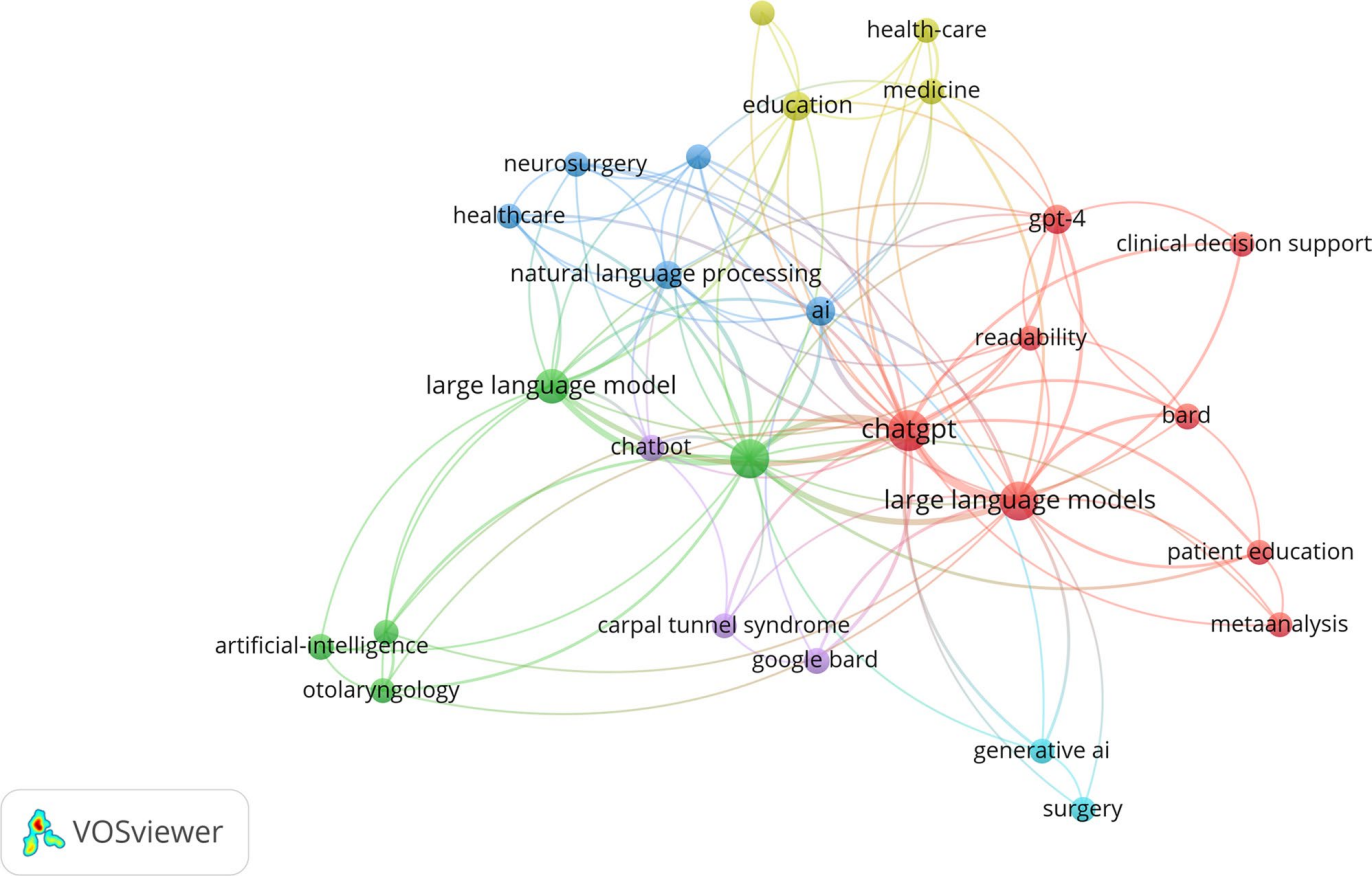
Keyword co-occurrence and trends analysis in the surgery field

A total of 706 articles were identified for surgery. The keyword co-occurrence demonstrated the close integration of LLM applications with surgical specialties, including neurosurgery and otolaryngology. Frequent terms included “patient education” (72 occurrences), ‘medical education’ (43), and ‘readability’ (32), reflecting LLM use for patient communication and educational resources (Fig. 10, Table 4).

**Table 4:** Top 10 highly cited publications in the surgery field

Rank	Title	Journal	First author	Year	Citations (total)
1	Assessing the accuracy of responses by the language model ChatGPT to questions regarding bariatric surgery	*Obesity Surgery*	Samaan JS	2023	214
2	Implications of large language models such as ChatGPT for dental medicine	*Journal of Esthetic and Restorative Dentistry*	Eggmann F	2023	187
3	Assessing ChatGPT responses to common patient questions regarding total hip arthroplasty	*Journal of Bone and Joint Surgery-American Volume*	Mika AP	2023	181
4	ChatGPT goes to the operating room: evaluating GPT-4 performance and its potential in surgical education and training in the era of large language models	*Annals of Surgical Treatment and Research*	Oh N	2023	151
5	Can ChatGPT be used in oral and maxillofacial surgery?	*Journal of Stomatology Oral and Maxillofacial Surgery*	Balel Y	2023	145
6	Performance of ChatGPT and GPT-4 on neurosurgery written board examinations	*Neurosurgery*	Ali R	2023	138
7	Aesthetic surgery advice and counseling from artificial intelligence: a rhinoplasty consultation with ChatGPT	*Aesthetic Plastic Surgery*	Xie Y	2023	138
8	ChatGPT is equivalent to first-year plastic surgery residents: evaluation of ChatGPT on the plastic surgery in-service examination	*Aesthetic Surgery Journal *	Humar P	2023	126
9	Comparison of ChatGPT-3.5, ChatGPT-4, and orthopaedic resident performance on orthopaedic assessment examinations	*Journal of the American Academy of Orthopaedic Surgeons*	Massey PA	2023	124
10	The transformative role of artificial intelligence in dentistry: a comprehensive overview. Part 1: fundamentals of AI, and its contemporary applications in dentistry	*International Dental Journal*	Samaranayake L	2025	111

Influential studies have highlighted both its potential and limitations. Samaan et al. (2023, *Obesity Surgery*, 214 citations) [19] reported 78% accuracy for ChatGPT in bariatric-surgery questions, decreasing to 52% for complex complications and necessitating physician oversight. Martin et al. (2023, *Journal of Bone and Joint Surgery-American Volume*, 181 citations) [20] showed that ChatGPT achieved 82% completeness in rehabilitation guidance for total hip arthroplasty, approaching junior-resident performance (85%).

#### Radiology, nuclear medicine and medical imaging field

**Fig. 11:**
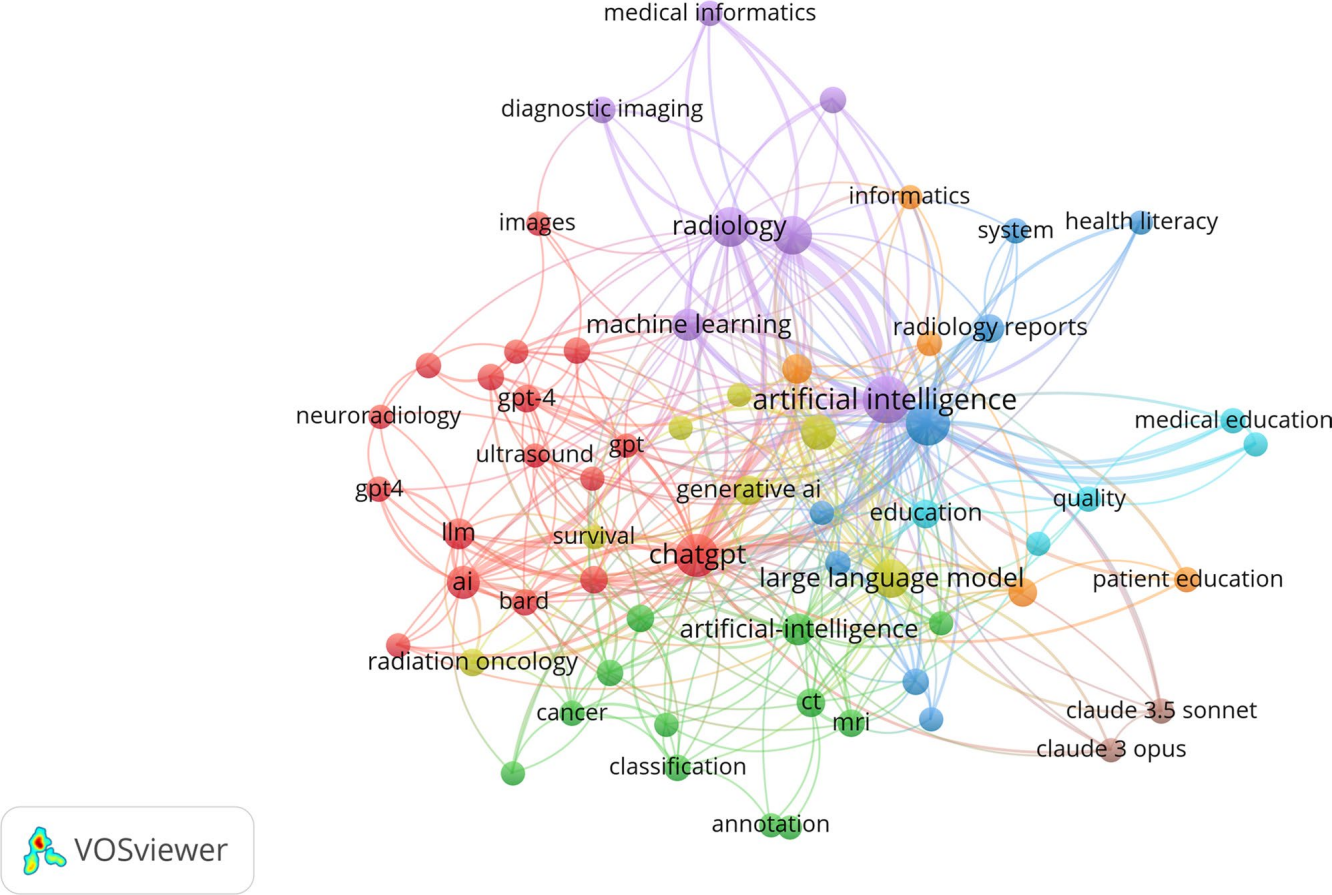
Keyword co-occurrence and trends analysis in the radiology, nuclear medicine and medical imaging field

This field contributed 289 articles. Keywords emphasized the integration of AI into diagnostic-imaging workflows, with the frequent co-occurrence of ‘radiology,’ ‘CT,’ “breast imaging,” and “lung cancer.” Terms such as “image segmentation,” “generative AI” (46 occurrences), and “radiology reports” (6 occurrences) reflected growing adoption of LLMs for automated reporting and workflow optimization (Fig. 11, Table 5).

**Table 5:** Top 10 highly cited publications in thr radiology, nuclear medicine and medical imaging field

Rank	Title	Journal	First author	Year	Citation (total)
1	ChatGPT makes medicine easy to swallow: an exploratory case study on simplified radiology reports	*European Radiology*	Jeblick K	2024	281
2	Revolutionizing radiology with GPT-based models: current applications, future possibilities and limitations of ChatGPT	*Diagnostic and Interventional Imaging*	Lecler A	2023	257
3	Potential of ChatGPT and GPT-4 for data mining of free-text CT reports on lung cancer	*Radiology*	Fink MA	2023	197
4	A context-based chatbot surpasses radiologists and generic ChatGPT in following the ACR appropriateness guidelines	*Radiology*	Rau A	2023	112
5	Potential use cases for ChatGPT in radiology reporting	*American Journal of Roentgenology*	Abou Elkassem A and Smith AD	2023	101
6	Feasibility of using the privacy-preserving large language model vicuna for labeling radiology reports	*Radiology*	Mukherjee P	2023	89
7	Decoding radiology reports: potential application of OpenAI ChatGPT to enhance patient understanding of diagnostic reports	*Clinical Imaging*	Li H	2023	88
8	Quantitative evaluation of large language models to streamline radiology report impressions: a multimodal retrospective analysis	*Radiology*	Doshi R	2024	79
9	The impact of large language models on radiology: a guide for radiologists on the latest innovations in AI	*Japanese Journal of Radiology*	Nakaura T	2024	76
10	Accuracy of ChatGPT generated diagnosis from patient’s medical history and imaging findings in neuroradiology cases	*Neuroradiology*	Horiuchi D	2024	75

Empirical studies underscored both the strengths and limitations of LLMs. Jeblick et al. (2024, *European Radiology*, 281 citations) [21] demonstrated that ChatGPT could simplify radiology reports to improve patient understanding, whereas Lecler et al. (2023, *Diagnostic and Interventional Imaging*, 257 citations) [22] showed that GPT-4 achieved 85% completeness in chest CT report generation; however, the accuracy for rare findings was limited to 61%. Fink et al. (2023, *Radiology*; 197 citations) [23] validated the utility of LLMs in mining free-text CT reports for lung cancer, highlighting opportunities to enhance radiological efficiency.

## Discussion

### Principal findings in context

Research expanded sharply after 2022, peaking between 2024 and 2025. Output and citations were concentrated in United States of America and European Union institutions, whereas China showed accelerated growth in 2024. Research emphasis clustered around United States Medical Licensing Examination (USMLE)/questions and answers, physician-chatbot comparisons, GPT‑4 evaluations, and radiology reporting. A shift from exam‑style accuracy studies toward patient‑facing communication and workflow drafting was noted. Evaluation reporting remained focused on accuracy, with limited attention being paid to fairness, toxicity, uncertainty, and resource metrics.

Keyword clusters emphasized “AI/LLM/ChatGPT/GenAI,” “readability/health literacy/patient communication,” and “machine learning/lung cancer/imaging.” A visible shift was observed toward patient-facing applications, particularly readability and communication [24, 25]. Cross-disciplinary citation flows underscored the interaction between technical innovations and clinical applications. These findings are consistent with prior reviews noting the rapid growth of LLM applications in summarization, documentation, and patient interaction, while concerns regarding bias, hallucination, privacy, and regulatory readiness persist [24, 26, 27]. Evaluations remain overly focused on accuracy metrics, with insufficient consideration given to fairness, toxicity, feasibility (e.g., latency and cost), and uncertainty calibration. To address these gaps, specialty landscapes have been reframed as actionable narratives linking bibliometric structures with near-term clinical applications under safety and governance constraints. These bibliometric signals should be interpreted as indicators of research attention and thematic momentum, rather than as direct evidence of clinical readiness, implementation maturity, or regulatory clearance. Accordingly, DECIDE-AI was used here as an interpretive scaffold for early-stage evaluation priorities, and not as a surrogate measure of deployment readiness.

The supplementary keyword analysis also highlighted the technological evolution of medical LLM research and its alignment with specialty-specific needs. Early work (2022–2023) focused on benchmarking general-purpose models and shifted toward clinical customization (2023–2024), followed by multimodal and workflow-oriented approaches after 2024. Technology adoption varied by specialty: the radiology field prioritized VLMs and RAG for imaging and reporting, the surgery field applied supervised education and perioperative communication, and the internal-medicine field adopted diverse technologies for complex clinical scenarios. This finding demonstrates that clinical requirements and data modalities, rather than technological advancements alone, drive the development of medical LLM applications.

The geographic concentration and limited reporting of fairness, uncertainty, and deployment metrics may reflect differences in the research infrastructure and policy attention across jurisdictions. However, bibliometric data do not directly measure regulatory maturity or implementation capacity. We interpret national health-AI policies as a contextual backdrop that may shape publication priorities rather than as causal explanations. United States of America has articulated device boundaries for clinical decision support via the Food and Drug Administration guidelines [28, 29], the European Union has established high-risk obligations under the AI act [30], and China’s GenAI measures have catalyzed rapid activity while prioritizing security assessments and data controls [31]. The regulatory convergence of documentation, continuous monitoring, and change control may provide a contextual background for publication priorities, rather than causal explanations.

### Domain-focused insights

To strengthen the translational interpretation, we structured the Discussion section using the DECIDE-AI framework, which emphasizes intended use, clinical workflow, foreseeable hazards, outcome measures, and monitoring needs [14].

### Medicine, general and internal

Bibliometric findings showed the strong visibility of communication-oriented themes, question-answering tasks, and health-literacy applications in this field. Most evaluations relied on exam-style datasets or synthetic vignettes with predominantly USMLE-style benchmarks [15]. Consistent with recent reviews, these studies prioritized accuracy while underassessing fairness, harm, and uncertainty calibration [18].

Within DECIDE-AI framework, clinical contexts include low-acuity portal messaging, discharge summaries, and supervised patient education. Its intended use is limited to draft generation with retrieval-augmented grounding. The anticipated hazards include outdated guidance, subtle misstatements, and equity-sensitive communication gaps that require escalation to clinician reviews. Early value includes readability adaptation, multilingual support, and supervised triage. Measures extend beyond accuracy to include guideline concordance, readability indices, comprehension verification, and equity/toxicity signals. Monitoring includes uncertainty-template adherence, audit logs, and regular bias/toxicity reviews.

#### Surgery

Surgical applications were most consistently linked to patient education, readability, and perioperative communication, supporting a near-term focus on supervised educational functions, rather than autonomous decision support. LLMs demonstrated reasonable accuracy for routine questions but underperformed in complex complication management, necessitating physician review [32]. These findings align with reviews suggesting documentation and education as near-term applications [32, 33].

Within DECIDE-AI, LLMs are confined to education and structured draft support with mandatory surgeon supervision. The anticipated risks include inappropriate advice on complications and consent omissions, which are mitigated by escalation protocols. Near-term applications include procedure-specific education, consent checklists, and personalized discharge summaries. Metrics prioritize consent completeness, comprehension verification, and time saving for clinicians. Monitoring processes emphasize scope enforcement, explicit AI disclosure, and incident capture.

#### Radiology, nuclear medicine and medical imaging

Research has focused on report generation, case summarization, and education, although the accuracy has declined for rare findings and nuanced descriptors [34]. This emphasis is aligned with our specialty analysis and supplementary technology-specific results.

DECIDE-AI alignment restricts the use of draft generation within structured templates, requiring a radiologist sign-off. The anticipated hazards include the misreporting of critical findings and unreliable comparisons. Mitigation includes anomaly flags and mandatory escalation. Short-term benefits include template-constrained drafts, patient-friendly summaries, and trainee feedback. These metrics include structural completeness, terminology adherence, and rare-finding sensitivity. Monitoring processes emphasize controlled knowledge bases, audit trails, and zero-tolerance thresholds for critical errors.

### Evaluation and governance agenda across domains

These findings highlight the need to move beyond accuracy as a sole benchmark. Evaluations should adopt multidimensional frameworks, including accuracy, fairness, toxicity, uncertainty calibration, deployment metrics, and real-world effectiveness [18, 33]. Standardized benchmarks using de-identified clinical artifacts are essential, along with model cards, monitoring dashboards, and failure-mode registries.

### Actionable recommendations for stakeholders

Clinicians and institutions can pilot low-risk applications tailored to their specialty needs. In internal medicine, these include RAG-based educational materials and supervised triage. In surgery, perioperative education can be standardized based on the surgeon’s review. In radiology, template-based report generation can be introduced under mandatory supervision. Health systems should prioritize transparency, biased audits, and privacy compliance [28]. Policymakers should fund benchmarks and incentivize multicenter evaluations.

### Limitations

This study has several limitations. First, WoSCC excludes many preprints and industry white papers, in which LLM advances typically debut. Second, English-only indexing under-represents non-English outputs. Third, the 2025 indexing and citation accrual lags bias results toward earlier years. Fourth, author/institution disambiguation may introduce misclassification. Fifth, network structures can vary with analytical thresholds. Sixth, bibliometric prominence does not equate to clinical readiness, as many deployments occur outside peer-reviewed journals.

## Conclusions

LLM research in healthcare is expanding rapidly with distinct specialty-specific trajectories. When applied to early-stage clinical evaluations, the DECIDE-AI framework highlights consistent low-risk pathways to deliver near-term values across medicine, general and internal; surgery; and radiology, nuclear medicine and medical imaging. Coupling these pathways with traceability, fairness, and deployment metrics and aligning monitoring with evolving regulatory frameworks may help translate the current developments into safe, equitable, and sustainable clinical adoption. 

## Supplementary Information

Below is the link to the electronic supplementary material.

Supplementary Material 1

## Data Availability

The data utilized in this review were sourced from the Web of Science databases, as indicated in the articles, images, and tables. The authors are not authorized to disseminate the data.

## Abbreviations

AI: Artificial intelligence
CAGR: Compound annual growth rate
DECIDE-AI: Developmental and exploratory clinical investigations of decision support systems driven by Artificial Intelligence
GenAI: Generative artificial intelligence
LLM: Large language model
PEFT: Parameter-efficient fine-tuning
RAG: Retrieval-augmented generation
USMLE: United States medical licensing examination
VLM: Vision-language model
WoSCC: Web of science core collection
