# Supplementary material for: Mapping the generative era: a bibliometric and specialty-focused analysis of large language models in healthcare
Source: Vis Comput Ind Biomed Art. 2026 Aug 14;9:16. doi: 10.1186/s42492-026-00228-y (PMC13476431; doi:10.1186/s42492-026-00228-y)
Supplement: Supplementary file 1 [file 42492_2026_228_MOESM1_ESM.docx]

**Mapping the Generative Era: A Bibliometric and Specialty-Focused Analysis of Large Language Models in Healthcare**

Supplementary Table 1. Top 10 highly cited conference publications in the field of large language models in healthcare

| Rank | Title | Journals | First author | Year | Citations (Total) |
| --- | --- | --- | --- | --- | --- |
| 1 | An artificial intelligence language model improves readability of burns first aid information | BURNS | Baldwin, AJ | 2024 | 13 |
| 2 | Evaluating ChatGPT as a patient resource for frequently asked questions about lung cancer surgery-a pilot study | JOURNAL OF THORACIC AND CARDIOVASCULAR SURGERY | Ferrari-Light, D | 2025 | 9 |
| 3 | Does using artificial intelligence take the person out of personal statements? We can't tell | SURGERY | Whitrock, JN | 2024 | 9 |
| 4 | De novo generation of colorectal patient educational materials using large language models: Prompt engineering key to improved readability | SURGERY | Ellison, IE | 2025 | 5 |
| 5 | Generated Responses to Routine Cervical Spine Surgery Questions | NEUROSURGERY | Yoseph, E | 2025 | 3 |
| 6 | A Comparative Assessment of ChatGPT vs Google Translate for the Translation of Patient Instructions | JOURNAL OF THE AMERICAN COLLEGE OF SURGEONS | Rao, P | 2024 | 3 |
| 7 | Using Large Language Models to Create Patient Centered Consent Forms | INTERNATIONAL JOURNAL OF RADIATION ONCOLOGY BIOLOGY PHYSICS | Beattie, J | 2024 | 3 |
| 8 | Constructing adverse event timelines for patients receiving CAR-T therapy using large language models | JOURNAL OF CLINICAL ONCOLOGY | Guillot, J | 2024 | 3 |
| 9 | Automating the detection of treatment progression in patients with lung cancer using large language models | JOURNAL OF CLINICAL ONCOLOGY | Sivarajkumar, S | 2024 | 3 |
| 10 | Can ChatGPT affect health literacy? | EUROPEAN JOURNAL OF PUBLIC HEALTH | Susnjara, IM | 2024 | 2 |
